# Identifying the association between single nucleotide polymorphisms in *KCNQ1*, *ARAP1*, and *KCNJ11* and type 2 diabetes mellitus in a Chinese population

**DOI:** 10.7150/ijms.48072

**Published:** 2020-08-29

**Authors:** Yiping Li, Keyu Shen, Chuanyin Li, Ying Yang, Man Yang, Wenyu Tao, Siqi He, Li Shi, Yufeng Yao

**Affiliations:** 1 Department of Endocrinology and Metabolism, The Second People's Hospital of Yunnan Province & The Fourth Affiliated Hospital of Kunming Medical University, Kunming 650021, Yunnan, China.; 2 Department of Medicine, Dentistry and Healthy Science, The University of Melbourne, Melbourne VIC3010, Australia.; 3 Institute of Medical Biology, Chinese Academy of Medical Sciences & Peking Union Medical College, Kunming 650118, Yunnan, China.; 4 School of Clinical Medicine, Dali University, Dali 671000, Yunnan, China.

**Keywords:** type 2 diabetes mellitus, single nucleotide polymorphisms

## Abstract

**Background:** Type 2 diabetes mellitus (T2DM) has a high global prevalence, and insufficient insulin secretion is one of the major reasons for its development. Therefore, investigating the association between T2DM and the single nucleotide polymorphisms (SNPs) in genes associated with insulin secretion is necessary.

**Methods:** T2DM (1,194) and nondiabetic (NDM) (1,292) subjects were enrolled and the ten single nucleotide polymorphisms (SNPs) in *KCNQ1*, *ARAP1*, and *KCNJ11* associated with insulin secretion were genotyped in a Chinese population.

**Results:** Our data revealed that the rs2237897T allele in *KCNQ1* is the protective allele for T2DM (*P<*0.001, OR=0.793; 95%CI: 0.705-0.893). However, the A allele of rs1552224 in *ARAP1* may be a risk factor for T2DM (*P=*0.002, OR=12.070; 95% CI: 1.578-92.337). The haplotype analysis revealed that rs151290-rs2237892CC and rs2237895-rs2237897CC in *KCNQ1* constitute the risk haplotype in T2DM development (*P=*0.010, OR=1.160; 95% CI: 1.037-1.299 and *P=*0.004, OR=1.192; 95% CI: 1.057-1.344). Moreover, rs2237895-rs2237897AT in *KCNQ1* constitutes the protective haplotype in T2DM (*P=*0.001, OR=0.819; 95% CI: 0.727-0.923). In the inheritance models analysis, the rs2283228 (C/A-C/C) genotype is the protective factor compared to the A/A genotype (*P=*0.005, OR=0.79; 95% CI: 0.68-0.93). For rs2237897, the C/T-T/T genotype is the protective factor compared to the C/C genotype (*P<*0.001, OR=0.74; 95% CI: 0.63-0.87). Furthermore, when compared with the rs2237897 (C/T-T/T) genotype, rs2237897C/C genotype showed higher HbA1C levels (8.731±2.697 vs 9.282±2.921, *P=*0.001).

**Conclusion:** Our results revealed that genetic variations in *KCNQ1* and *ARAP1* were associated with T2DM susceptibility in a Chinese population.

## Introduction

The prevalence of diabetes in adults 20-79 years from mainland China is dramatically increasing and has reached 10.9% (https://diabetesatlas.org/en/), according to the latest data of the International Diabetes Federation Atlas (Ninth edition 2019). In fact, the number of adults with diabetes in China was 116.4 million and ranked the first in the world in 2019 (https://diabetesatlas.org/en/). Type 2 diabetes mellitus (T2DM) accounts for approximately 85-95% of all cases of diabetes, which is a progressive hyperglycemic disease initially characterized by decreasing sensitivity of peripheral tissues to plasma insulin, accompanied by compensatory hyperinsulinemia, and a gradual failure of the pancreatic β-cells to maintain glucose homeostasis 1. Insufficient insulin secretion and insulin resistance are associated with the pathophysiology of T2DM development. Compared to the European or African populations, the Chinese population, as one of the East Asian populations, exhibits signs of lower insulin secretion, apart from insulin resistance, which indicates that the insulin secretion function of pancreatic β cells is a critical factor associated with the development of T2DM in the Chinese population 2.

Insulin secretion by the pancreatic β cells comprises of a series of dynamic ion exchanges. The closure of the ATP-dependent potassium (KATP) channel increases the K^+^ concentration in pancreatic β cells, following which the influx of Ca^2+^ into the pancreatic β cells stimulates insulin release 3. The *KCNJ11* (potassium inwardly rectifying channel, subfamily J, member 11), *KCNQ1* (potassium voltage-gated channel, KQT-like subfamily, member 1), and *ARAP1* encode proteins involved in insulin secretion and all of them are expressed in the pancreatic islets 3-5. *KCNJ11* is located at 11p15.1 6 and encodes the inward rectifier potassium ion channel (Kir6.2) which forms a classic KATP channel by binding to the sulfonylurea receptor 7. *KCNQ1* is located at 11p15.5 and encodes the pore-forming α subunit of the KvLQT1 channel that is responsible for the repolarization of the pancreatic β-cell membrane and downregulates insulin secretion by blocking Ca^2+^ influx 8. *ARAP1* is located at 11q13.4 and encodes ARAP1 that might play a role in reducing insulin secretion and increasing the risk of T2DM 9.

Since 2003, several studies have revealed that the single nucleotide polymorphisms (SNPs) in *KCNQ1*, *ARAP1*, and *KCNJ11* are associated with insulin secretion and/or T2DM in different populations; however, the results from such studies are varied 10-17. The inconsistency in the results suggests that the association between T2DM and the variants of *KCNQ1*, ARAP1, and *KCNJ11* differ in different populations. For example, in 2008, Unoki et al. reported that the *KCNQ1* rs2237897 genotype was associated with T2DM in Japanese, Singaporean, and Danish populations 16. However, Yasuda et al. did not observe any association between rs2237897 and T2DM in a Japanese population 17.

The current study aims to investigate the genetic effects of the polymorphisms in *KCNQ1*, *ARAP1*, and *KCNJ11* on T2DM in a Chinese population. Therefore, the ten SNPs in the three genes (rs151290, rs163184, rs2237892, rs2237895, rs2237897, rs2283228, and rs231362 in *KCNQ1*, rs1552224 in *ARAP1*, and rs5210 and rs5219 in *KCNJ11*) and the risk of T2DM development were investigated simultaneously in a Chinese population to determine their association.

## Materials and Methods

### Ethics statement

The Institutional Review Board of the Second People's Hospital of Yunnan Province approved the study before the commencement of the investigation. Moreover, the protocol adopted in this investigation was in accordance with the principles stated in the Helsinki Declaration of 1975, revised in 2008. All participants provided written informed consent.

### Subjects

The subjects recruited in this study included 1,194 patients of T2DM (762 males and 432 females) who were diagnosed at the Second People's Hospital of Yunnan Province between February 2017 and August 2019. The diagnostic standard of T2DM was in accordance with the World Health Organization criteria published in 1999 and American Diabetes Association (ADA) guidelines in 2020^18^. In detail, the 75-g oral glucose tolerance test were done in all the T2DM subjects to confirm the diagnosis of diabetes. Simultaneously, insulin secretion and C peptides level were measured to exclude subjects with type 1 diabetes. For the subjects with the age below 40 years old, islet cell autoantibodies and glutamic acid decarboxylase autoantibodies were detected to exclude autoantibody positive subjects. Gestational women were not included. Specific types of diabetes were excluded by history illness, such as glucocorticoid induced diabetes. The NDM group included 1,292 subjects (790 males and 502 females) with no family history of diabetes mellitus, who were recruited from among individuals undergoing routine health checkup at the Second People's Hospital of Yunnan Province. Subjects with impaired glucose tolerance and/or glycosylated hemoglobin (HbA1C) levels above 5.7% were excluded from the NDM group 18. In addition, subjects with hypertension or coronary heart disease were also excluded from the study. All participants (T2DM and NDM) self-reported to be of Han ethnicity.

### Laboratory measurements

Venous blood samples were collected the morning after the subjects had fasted for 12 hours. Fasting plasma glucose (FPG) was assayed using the Glucose Kit (Hexokinase method). The levels of total cholesterol (TC) and the triglycerides (TG) were measured by total cholesterol and triglycerides Kits (Chod-Pap and Gpo-Pap Method), respectively. The high-density lipoprotein cholesterol (HDL-C) and low-density lipoprotein cholesterol (LDL-C) was directly assayed using the Cholestest N HDL and LDL Kits (Direct method), respectively. HbA1C was measured by Norudia N HbA1c Kit (Enzymatic method). All laboratory measurements were performed using a HITACHI 7600-020 Automatic Analyzer.

### Selection and genotyping of SNPs

Based on the literatures 10-17, we primarily focused on the three genes which were shown to exert effects on insulin secretion by pancreatic islets. The six SNPs from *KCNQ1* (rs151290, rs163184, rs2237892, rs2237895, rs2237897, and rs2283228) were located in intron 15, and the rs231362 was located in intron 11. The rs5210 and rs5219 in *KCNJ11* were located in the 3'UTR and exon 1, respectively. The rs1552224 was located in the 5'UTR of *ARAP1*.

The QIAamp Blood Mini Kit (Qiagen, Hilden, Germany) was used to extract genomic DNA from peripheral lymphocytes. The ten SNPs from the three genes (rs151290, rs163184, rs2237892, rs2237895, rs2237897, rs2283228, and rs231362 in *KCNQ1*, rs1552224 in *ARAP1*, and rs5210 and rs5219 in *KCNJ11*) were genotyped using the MassARRAY Analyzer 4.0 (Agena, Inc). The PCR primers were designed using the AssayDesigner 3.1 (Sequenom lnc., San Diego, CA, USA). Four microliters of the PCR master mix was added into the reaction wells with 1 μl template DNA (25 ng/μl) in a 384-well plate. The PCR reaction conditions were the same as those in our previous study 19. The PCR products were treated with 2 μl shrimp alkaline phosphatase (SAP) per-well to remove the dNTP; the reaction conditions were set at: 37 °C for 20 min and 85 °C for 5 min. Next, 2 μl of the EXTEND Mix was added for single base extension using the following PCR cycle conditions: 1) 94 °C for 30 sec, 2) 94 °C for 5 sec, 3) 52 °C for 5 sec, 4) 80 °C for 5 sec, 5) 72-94 °C for 3 min, and the steps 2-4) were repeated for 40 cycles with 5 repetitions of steps 3) and 4) per cycle. The 9 μl reaction products were subjected to resin purification, and the final products were transferred to a 384-well SpectroCHIP bioarray by MassARRAY Nanodispenser RS1000 machine (Agena, Inc, San Diego, CA, USA). The MALDI-TOF mass spectrometer (Agena, Inc) was used to read the SpectroCHIP and the raw genotyping data was obtained using the TYPER 4.0 software.

### Statistical analysis

The ages, glucose, and lipid metabolic parameters (TC, HDL-C, TG, LDL-C, FPG, and HbA1C) between the T2DM and control groups were compared using the Student's t-test. The gender distribution between the T2DM and control groups was compared using the Chi-square test. All polymorphic loci were tested for deviation from the Hardy-Weinberg equilibrium in the control group with a threshold of 0.05. Basic statistical analysis for the association between the alleles, genotypes, haplotypes, and disease were performed using the SHEsis software 20, 21. Risks were estimated by the odds ratio (OR) with 95% confidence interval (95%CI). Linkage disequilibrium (LD) among the SNPs was also estimated, where the LD coefficient “D” was calculated using the SHEsis software 20, 21. LD is presented as “pairwise D'”, with the D' values defined in the range 1, with a value of “1” indicating perfect disequilibrium. A D' value above 0.8 indicated the existence of different loci in the LD. The distribution and differences in the haplotypes between the case and control groups were determined using the SHEsis software and the haplotype frequency <0.03 was ignored during the analysis 20, 21. The association between T2DM and each SNP was analyzed for the mode of inheritance using the SNPStats software 22. The Akaike information criterion (AIC) and the Bayesian information criterion (BIC) were used to determine the best fit model for each SNP. The glucose and lipid metabolic parameters of different genotypes of each SNP were compared using the one-way ANOVA and comparations between the two groups were tested using the Bonferroni correction. The Student's *t*-test and Chi-square test were performed using the SPSS 21 (Chicago, IL). The statistical power was calculated using PS Software 23. The significant threshold after Bonferroni correction for multiple comparisons was indicated by *P<*0.005 (0.05/10) for each SNP.

## Results

### Subject characteristics

Table 1 lists the clinical characteristics and the glucose and lipid metabolic parameters of the enrolled subjects. There were no significant differences in age or gender between the subjects from the T2DM and NDM groups, or in age between the male and female subjects from both groups (P>0.05). However, there were significant differences in the glucose and lipid metabolic parameters (TC, TG, HDL-C, LDL-C, FPG, and HbA1C levels) between subjects from the T2DM and NDM groups (*P<*0.001) (Table 1). Notably, compared to the subjects from the NDM group, the T2DM subjects exhibited dyslipidemia, that is, higher levels of TC, TG, and LDL-C and lower levels of HDL-C.

### Association between the SNPs and T2DM

The assessment of the genotype frequencies of the ten SNPs in the NDM group indicated that these were in Hardy-Weinberg equilibrium (P>0.05) (Table 2). The allele and genotype frequencies of the ten SNPs in the three genes in both the T2DM and NDM groups are listed in Table 2. Two SNPs (rs2237897 in *KCNQ1* and rs1552224 in *ARAP1*) had significantly different genotype frequencies in the T2DM and NDM groups (*P<*0.005) (Table 2). The alleles of two SNPs (rs2237897 in *KCNQ1* and rs1552224 in *ARAP1*) displayed significant differences in distribution between the NDM and T2DM groups (Table 2). For the rs2237897 in *KCNQ1*, the T allele is the protective allele against T2DM (*P<*0.001, OR=0.793; 95%CI: 0.705-0.893). The A allele of rs1552224 in *ARAP1* was associated with T2DM and functioned as a risk allele in the development of T2DM (*P=*0.002, OR=12.070; 95%CI: 1.578-92.337) (Table 2). The other eight SNPs, including the alleles and genotypes of rs2237892, rs2237895, rs151290, rs163184, and rs231362 in *KCNQ1*, rs1552224 in *ARAP1*, and rs5210 and rs5219 in *KCNJ11*, did not exhibit any association with T2DM (Table 2).

### Association of the haplotypes of the SNPs with T2DM

The LD of the six SNPs (rs151290, rs2237892, rs163184, rs2283228, rs2237895, and rs2237897) in *KCNQ1* intron 15 was estimated. According to the D' value (D'>0.8), the three haplotypes - rs151290-rs2237892, rs2237892-rs163184-rs2283228, and rs2237895-rs2237897 - were constructed. The haplotype analysis revealed that the frequency distributions of the rs151290-rs2237892CC and rs2237895-rs2237897CC haplotypes in the T2DM and NDM groups had statistically significant differences (*P=*0.010 and *P=*0.004) (Table 3). The rs151290-rs2237892CC (OR=1.160; 95% CI: 1.037-1.299) and rs2237895-rs2237897CC (OR=1.192; 95% CI: 1.057-1.344) were associated with a greater risk of T2DM development (Table 3). Conversely, the rs2237895-rs2237897AT haplotype was associated with a lower risk of T2DM development (*P=*0.001, OR=0.819; 95% CI: 0.727-0.923). There was no association between the other two haplotypes (rs151290-rs2237892 and rs2237892-rs163184-rs2283228) and T2DM. Although rs5210 and rs5219 in *KCNJ11* were in LD (D'=0.924), the constructed haplotypes were not associated with T2DM (*P*>0.05).

### Mode of inheritance analysis

The inheritance model (codominant, dominant, recessive, overdominant, and log-additive) of these SNPs was constructed using SNPStats 22. The best fit inheritance model with the lowest AIC for both rs2237897 and rs2283228 in *KCNQ1* was dominant. For rs2237897, compared to the C/C genotype, the (C/T-T/T) genotype was the protective factor against T2DM (*P<*0.001, OR=0.74; 95% CI: 0.63-0.87) (Table 4). For rs2283228, the (C/A-C/C) genotype was the protective factor compared to the A/A genotype (*P=*0.005, OR=0.79; 95% CI: 0.68-0.93) (Table 5). The other SNPs did not exhibit any association with T2DM in the given population (Supplementary Table 1-7).

### Association between the genotypes of the ten SNPs and the metabolic phenotypes

In the NDM group, there were no significant differences of the glucose and lipid metabolic parameters among the genotypes of these SNPs, including FPG, TC, HDL-C, TG, LDL-C, and HbA1C levels (data not shown). However, in the T2DM group, the HbA1C levels showed a significant difference among C/C, C/T and T/T genotypes of rs2237897 (*P=*0.003) (Supplementary Table 8). The HbA1C levels was 9.282±2.921 (%) in C/C genotype, 8.688±2.674 (%) in C/T and 8.949±2.817 (%) in T/T. When compared with the rs2237897 (C/T-T/T) genotype, rs2237897C/C genotype showed higher HbA1C levels (9.282±2.921 vs 8.731±2.697, *P=*0.001). The other glucose and lipid metabolic parameters showed no significant among the genotypes of these SNP in T2DM group (*P*>0.05) (data not shown).

## Discussion

*KCNJ11*, *KCNQ1*, and *ARAP1* play a critical role in the insulin secretion from pancreatic β cells. In the current study, we observed that the rs2237897 and rs2283228 in *KCNQ1* and the rs1552224 in *ARAP1* were associated with T2DM in a Chinese Han population.

*KCNQ1* encodes a pore-forming α subunit of the KvLQT1 channel that is involved in the insulin secretion mediated by Ca^2+^ dynamics 8. In 2008, Unoki et al. reported that the rs2237897C allele was associated with T2DM risk in a European population as well as in Asian populations 16. Following this, Tan et al. also reported that the rs2237897C allele was identified as a risk factor for T2DM development in a combined population, which included Chinese, Malay, and Asian Indian individuals 15. However, in 2008, Yasuda et al. reported no significant association between the rs2237897 and T2DM in a Japanese population 17. In the current study, we observed that the rs2237897C allele and the C/C genotype could pose as risk factors in the development of T2DM, which is consistent with the findings of a recent study by Qian et al., wherein they reported that the rs2237897C allele was associated with an increased risk of T2DM in a Chinese Han population 24. Notably, the frequency of rs2237897C, which was reported to be the risk allele in the German, Danish, and in the studied Chinese population, was different for the Chinese and the European populations (German 96.3%, Danish 97.0%, and Chinese 64.4-69.5%) 16, 25. In the current study, we also observed that the rs2237897C/C genotype could be associated with higher HbA1C levels in the T2DM group in the Chinese population, which indicates that the rs2237897C influenced the HbA1C levels in the T2DM group through the insulin secretory function. In 2009, Tan et al. reported that the rs2237897C allele was significantly associated with reduced corrected insulin response, a measure of β-cell insulin secretion function in the Chinese population 15. Concurrently, Mussig et al. also reported the association between the rs2237897C/C genotype and lower fasting insulin levels in a German population with high T2DM risk 25. These results indicate that the rs2237897 in *KCNQ1* could alter the ion dynamics in the KvLQT1 channel, thereby reducing insulin secretion and leading to hyperglycemia 15.

The association between T2DM and the rs2283228A allele in *KCNQ1* and was first reported in Japanese and Danish populations, while it was not reported in Singaporean populations 16. In 2009, Tan et al. reported that the rs2283228A allele was also associated with T2DM in a combined Asian population, including Chinese, Malay, and Asian Indian populations 15. In the current study, our results indicated that in T2DM development, the rs2283228(C/A-C/C) genotype was the protective factor compared to the A/A genotype. Moreover, Tan et al. reported that the rs2283228A allele was also associated with reduced insulin secretion in a Chinese population, which indicates that the rs2283228A might hinder pancreatic β-cell function and insulin production by altering the functioning of the KvLQT1 channel 15. Next, Hanson et al. reported the association between rs2283228A and lower insulin secretion based on intravenous glucose-induced insulin response in 288 normoglycemic American Indian individuals 26. Therefore, these results indicate that the role of *KCNQ1* rs2283228 in T2DM development could be mediated through Ca^2+^ dynamics, and subsequently affect insulin secretion.

In the current study, we observed that the distribution of the rs151290-rs2237892CC and rs2237895-rs2237897CC haplotypes was associated with a higher risk of T2DM development. Conversely, the rs2237895-rs2237897AT haplotype was associated with a lower risk of T2DM development. Since the T allele of the rs2237897 in *KCNQ1* acts as the protective allele against T2DM and the rs2237895-rs2237897 are in LD, the rs2237897 haplotype was associated with the development of T2DM. Notably, our results showed that rs151290-rs2237892CC was associated with a higher risk of T2DM development. However, we did not observe the association between the rs151290 and rs2237892 and development of T2DM prior to Bonferroni correction (*P=*0.100 and 0.120, respectively). Several studies have reported that the rs151290 and rs2237895 genotypes are associated with T2DM development in Asian and European populations 16, 17, 25, 27, 28. This discrepancy could be attributed to the relatively medium sample size, which is one of the limitations of the current study. Therefore, more samples should be studied in the future.

*ARAP1* encodes proteins associated with insulin secretion and is expressed in the pancreatic islets 4. The association of the rs1552224A allele with T2DM risk was reproducible in European populations 29, as well as in Asian populations, including a Chinese population 30. In the current study, our results also confirmed the association of the rs1552224A allele with T2DM in a Chinese population. In 2017, Carrat et al. reported that the rs1552224C allele was associated with the overexpression of the steroidogenic acute regulatory protein related lipid transfer domain protein 10 (STARD10). Additionally, the reduction in STARD10 levels, and not the increase in ARAP1 levels, was responsible for the impaired insulin secretion in pancreatic β cells, suggesting that the protective role of the rs1552224C allele in T2DM might be mediated by the overexpression of STARD10 and the subsequent improvement in glucose-induced Ca^2+^ dynamics and insulin secretion in pancreatic β cells 31.

There are varying reports of the association between T2DM and the rs5219 and rs5210 genotypes in *KCNJ11* in different populations 11, 32-39. Moreover, this association has yielded different results in the same population as well. For example, in 2009, Zhou et al. reported that compared to the rs5219 genotype (T/C+C/C), the rs5219T/T genotype was associated with a greater risk of T2DM in a Chinese population 34. However, in 2015, Qian et al. reported that there was no association between rs5219 and T2DM in another Chinese population 24. In the current study, we failed to observe any association of T2DM with rs5210 and rs5219. This discrepancy might have occurred since the effect of the allele in impaired insulin secretion was compensated by the insulin action, since the coexistence of insulin secretion deficiency and increased insulin action was observed in NGT adults with the rs5219 T/T genotype 7, 40. Therefore, the direct or indirect effects of rs5219 on insulin action should be elucidated in future studies.

A relatively moderate sample size may limit the statistical power of our study. The statistical power for the effect of rs2237897 in KCNQ1 and the rs1552224 in ARAP1 were calculated using PS software 23, and we found that our sample reached 0.848 and 0.602 of statistical efficacy respectively. In addition, the other limitation of the current study was that complications of T2DM was not included in the data derived from the T2DM individuals in the current study, and we could not analyze our results with complications of T2DM. Thus, a larger population should be investigated and the role of these SNPs on complications of T2DM should be elucidated in the future studies.

## Conclusions

Our study investigated the influence of the ten SNPs in KCNQ1, ARAP1, and KCNJ11 (rs151290, rs163184, rs2237892, rs2237897, rs2283228 and rs231362 in KCNQ1, rs1552224 in ARAP1, and rs5210 and rs5219 in KCNJ11), which influence insulin secretion by mediating ion dynamics, on T2DM in a Chinese population. The results indicated that the rs2237897 and rs2283228 in KCNQ1 and the rs1552224 in ARAP1 are associated with T2DM in the Chinese population we studied, suggesting that the functional effects of these variants associated with the insulin secretion ion channel need to be investigated further.

## Supplementary Material

Supplementary table S1.

## Figures and Tables

**Table 1:** Clinical characteristics and glucose and lipid metabolic parameters of the subjects enrolled in the present study

	Nondiabetic subject	Type 2 diabetes	*P* value
N	1292	1194	
Age (years)	52.707±10.520	52.490±12.101	0.634
Sex (M/F)	790/502	762/432	0.172
Age (M, years)	51.713±10.537	51.366±12.399	0.554
Age (F, years)	54.273±10.309	54.472±11.300	0.780
Total cholesterol (mmol/L)	4.521±1.050	4.816±1.076	<0.001
Triglycerides (mmol/L)	1.761±1.354	2.459±2.120	<0.001
High-density lipoprotein-cholesterol (mmol/L)	1.275±0.348	1.093±0.281	<0.001
Low-density lipoprotein-cholesterol (mmol/L)	2.629±0.848	2.794±0.969	<0.001
Fasting plasma glucose (mmol/L)	5.007±0.525	7.895±2.503	<0.001
HbA1C (%)	5.138±0.354	8.990±2.817	<0.001

**Table 2:** Comparison of genotypic and allelic distribution of seven SNPs (rs151290, rs2237892, rs163184, rs2283228, rs2237895, rs2237897 and rs231362) in KCNJ1, rs1552224 in ARAP1 and two SNPs (rs5210 and rs5219) in KCNQ1 between NDM and T2DM group

SNPs	Allele		Chi^2^	*P* value	Odds Ratio (95%CI)				Chi^2^	*P* value	H-W
rs151290	C (freq)	A (freq)				C/C (freq)	C/A(freq)	A/A(freq)			
T2DM	1495 (0.626)	893 (0.374)	2.703	0.100	1.101 (0.982-1.234)	466(0.390)	563(0.472)	165(0.138)	4.053	0.132	0.807
NDM	1559 (0.603)	1025 (0.397)				454(0.351)	651(0.504)	187(0.145)			0.058
rs2237892	C (freq)	T (freq)				C/C(freq)	C/T(freq)	T/T(freq)			
T2DM	1667 (0.698)	721 (0.302)	2.414	0.120	1.100 (0.975-1.240)	579(0.485)	509(0.426)	106(0.089)	2.740	0.254	0.696
NDM	1751 (0.678)	833 (0.322)				584(0.452)	583(0.451)	125(0.097)			0.238
rs163184	A (freq)	C (freq)				A/A(freq)	A/C(freq)	C/C(freq)			
T2DM	1321 (0.553)	1067 (0.447)	1.933	0.165	0.924 (0.826-1.033)	386(0.323)	549(0.460)	259(0.217)	5.711	0.058	0.016
NDM	1480 (0.573)	1104 (0.427)				421(0.326)	638(0.494)	233(0.180)			0.747
rs2283228	C (freq)	A (freq)				C/C(freq)	C/A(freq)	A/A(freq)			
T2DM	784 (0.328)	1604 (0.672)	6.03	0.014	0.863 (0.768-0.971)	130(0.109)	524(0.439)	540(0.452)	7.970	0.019	0.864
NDM	934 (0.361)	1650 (0.639)				154(0.119)	626(0.485)	512(0.396)			0.074
rs2237895	A (freq)	C (freq)				A/A(freq)	A/C(freq)	C/C(freq)			
T2DM	1586 (0.664)	802 (0.336)	5.172	0.023	0.871 (0.773-0.981)	509(0.426)	568(0.476)	117(0.098)	7.495	0.024	0.022
NDM	1794 (0.694)	790 (0.306)				621(0.481)	552(0.427)	119(0.092)			0.817
rs2237897	T (freq)	C (freq)				T/T(freq)	C/T(freq)	C/C(freq)			
T2DM	729 (0.305)	1659 (0.695)	14.643	<0.001	0.793 (0.705-0.893)	104(0.087)	521(0.436)	569(0.477)	15.632	<0.001	0.321
	921 (0.356)	1663 (0.644)				150(0.116)	621(0.481)	521(0.403)			0.087
rs231362	C (freq)	T (freq)				C/C(freq)	C/T(freq)	T/T(freq)			
T2DM	2116 (0.886)	272 (0.114)	0.942	0.506	0.942 (0.789-1.124)	940(0.787)	236(0.198)	18(0.015)	0.509	0.775	0.472
NDM	2305 (0.892)	279 (0.108)				1032(0.799)	241(0.187)	19(0.015)			0.255
rs1552224	A (freq)	C (freq)				A/A(freq)	A/C(freq)	C/C(freq)			
T2DM	2387 (1.000)	1 (0.000)	9.402	0.002	12.070 (1.578-92.337)	1193(0.999)	1(0.001)	0	9.429	0.002	0.988
NDM	2571 (0.995)	13 (0.005)				1279(0.990)	13(0.010)	0			0.856
rs5210	G (freq)	A (freq)				G/G(freq)	G/A(freq)	A/A(freq)			
T2DM	1148 (0.481)	1240 (0.519)	1.735	0.188	1.078 (0.964-1.205)	273(0.229)	602(0.504)	319(0.267)	1.804	0.406	0.733
NDM	1194 (0.462)	1390 (0.538)				270(0.209)	654(0.506)	368(0.285)			0.512
rs5219	C (freq)	T (freq)				C/C(freq)	C/T(freq)	T/T(freq)			
T2DM	1434 (0.601)	954 (0.399)	0.053	0.817	0.987 (0.881-1.105)	431(0.361)	572(0.479)	191(0.160)	0.628	0.731	0.958
NDM	1560 (0.604)	1024 (0.396)				462(0.358)	636(0.492)	194(0.150)			0.301

**Table 3:** The haplotype analysis for SNPs located in KCNQ1 gene intron 15 between NDM and T2DM group

haplotypes	D'	r^2^	T2DM (freq)	NDM (freq)	Chi^2^	*P* value	Odds Ratio (95%CI)
rs151290-rs2237892CC	0.813	0.479	1425.96 (0.597)	1449.30 (0.561)	6.692	0.010	1.160 (1.037-1.299)
rs2237895-rs2237897CC	0.945	0.209	801.95 (0.336)	761.10 (0.295)	8.225	0.004	1.192 (1.057-1.344)
rs2237895-rs2237897AT	0.945	0.209	728.95 (0.305)	892.10 (0.345)	10.786	0.001	0.819 (0.727-0.923)

**Table 4:** Different inheritance models analysis of the SNP rs2237897 between the NDM and T2DM group

Model	Genotype	NDM	T2DM	OR (95% CI)	*P* value	AIC	BIC
Codominant	C/C	521 (40.3%)	569 (47.6%)	1.00	0.001	3435.2	3464.3
	C/T	621 (48.1%)	521 (43.6%)	0.77 (0.65-0.91)			
	T/T	150 (11.6%)	104 (8.7%)	0.64 (0.48-0.84)			
Dominant	C/C	521 (40.3%)	569 (47.6%)	1.00	<0.001	3435.0	3458.3
	C/T-T/T	771 (59.7%)	625 (52.4%)	0.74 (0.63-0.87)			
Recessive	C/C-C/T	1142 (88.4%)	1090 (91.3%)	1.00	0.018	3442.9	3466.2
	T/T	150 (11.6%)	104 (8.7%)	0.73 (0.56-0.95)			
Overdominant	C/C-T/T	671 (51.9%)	673 (56.4%)	1.00	0.026	3443.5	3466.8
	C/T	621 (48.1%)	521 (43.6%)	0.84 (0.71-0.98)			
Log-additive	---	---	---	0.79 (0.70-0.89)	<0.001	3433.4	3456.7

**Table 5:** Different inheritance models analysis of the SNP rs2283228 between the NDM and T2DM group

Model	Genotype	NDM	T2DM	OR (95% CI)	*P* value	AIC	BIC
Codominant	A/A	512 (39.6%)	540 (45.2%)	1.00	0.019	3442.5	3471.6
	C/A	626 (48.5%)	524 (43.9%)	0.79 (0.67-0.94)			
	C/C	154 (11.9%)	130 (10.9%)	0.80 (0.62-1.04)			
Dominant	A/A	512 (39.6%)	540 (45.2%)	1.00	0.005	3440.5	3463.8
	C/A-C/C	780 (60.4%)	654 (54.8%)	0.79 (0.68-0.93)			
Recessive	A/A-C/A	1138 (88.1%)	1064 (89.1%)	1.00	0.420	3447.8	3471.1
	C/C	154 (11.9%)	130 (10.9%)	0.90 (0.70-1.16)			
Overdominant	A/A-C/C	666 (51.5%)	670 (56.1%)	1.00	0.023	3443.3	3466.6
	C/A	626 (48.5%)	524 (43.9%)	0.83 (0.71-0.98)			
Log-additive	---	---	---	0.86 (0.76-0.97)	0.013	3442.3	3465.6
