# Supplementary material for: Identifying the association between single nucleotide polymorphisms in KCNQ1, ARAP1, and KCNJ11 and type 2 diabetes mellitus in a Chinese population
Source: Int J Med Sci. 2020 Aug 29;17(15):2379–86. doi: 10.7150/ijms.48072 (PMC7484634; doi:10.7150/ijms.48072)
Supplement: Supplementary file 1 — Supplementary table S1. [file ijmsv17p2379s1.pdf]

Supplementary Table 1. Different inheritance models analysis of the rs151290 between the NDM and T2DM group.

Model	Genotype	NDM	T2DM	OR (95% CI)	*P* value	AIC	BIC
Codominant	C/C	454 (35.1%)	466 (39%)	1.00	0.130	3446.4	3475.5
	C/A	651 (50.4%)	563 (47.1%)	0.84 (0.71-1.00)			
	A/A	187 (14.5%)	165 (13.8%)	0.86 (0.67-1.10)			
Dominant	C/C	454 (35.1%)	466 (39%)	1.00	0.044	3444.4	3467.7
	C/A-A/A	838 (64.9%)	728 (61%)	0.85 (0.72-1.00)			
Recessive	C/C-C/A	1105 (85.5%)	1029 (86.2%)	1.00	0.640	3448.2	3471.5
	A/A	187 (14.5%)	165 (13.8%)	0.95 (0.76-1.19)			
Overdominant	C/C-A/A	641 (49.6%)	631 (52.9%)	1.00	0.110	3445.9	3469.1
	C/A	651 (50.4%)	563 (47.1%)	0.88 (0.75-1.03)			
Log-additive	---	---	---	0.91 (0.81-1.02)	0.094	3445.7	3468.9

Supplementary Table 2. Different inheritance models analysis of the SNP rs2237892 between the NDM and T2DM group.

Model	Genotype	NDM	T2DM	OR (95% CI)	*P* value	AIC	BIC
Codominant	C/C	584 (45.2%)	579 (48.5%)	1.00	0.260	3447.8	3476.9
	C/T	583 (45.1%)	509 (42.6%)	0.88 (0.75-1.04)			
	T/T	125 (9.7%)	106 (8.9%)	0.86 (0.65-1.14)			
Dominant	C/C	584 (45.2%)	579 (48.5%)	1.00	0.100	3445.8	3469.1
	C/T-T/T	708 (54.8%)	615 (51.5%)	0.88 (0.75-1.03)			
Recessive	C/C-C/T	1167 (90.3%)	1088 (91.1%)	1.00	0.510	3448	3471.3
	T/T	125 (9.7%)	106 (8.9%)	0.91 (0.70-1.20)			
Overdominant	C/C-T/T	709 (54.9%)	685 (57.4%)	1.00	0.210	3446.9	3470.2
	C/T	583 (45.1%)	509 (42.6%)	0.90 (0.77-1.06)			
Log-additive	---	---	---	0.91 (0.80-1.03)	0.120	3446.1	3469.4

Supplementary Table 3. Different inheritance models analysis of the rs163184 between the NDM and T2DM group.

Model	Genotype	NDM	T2DM	OR (95% CI)	*P* value	AIC	BIC
Codominant	A/A	421 (32.6%)	386 (32.3%)	1.00	0.060	3444.9	3473.9
	A/C	638 (49.4%)	549 (46%)	0.94 (0.79-1.13)			
	C/C	233 (18%)	259 (21.7%)	1.21 (0.97-1.52)			
Dominant	A/A	421 (32.6%)	386 (32.3%)	1.00	0.870	3448.4	3471.7
	A/C-C/C	871 (67.4%)	808 (67.7%)	1.01 (0.86-1.20)			
Recessive	A/A-A/C	1059 (82%)	935 (78.3%)	1.00	0.023	3443.3	3466.6
	C/C	233 (18%)	259 (21.7%)	1.26 (1.03-1.53)			
Overdominant	A/A-C/C	654 (50.6%)	645 (54%)	1.00	0.097	3445.7	3469
	A/C	638 (49.4%)	549 (46%)	0.87 (0.75-1.02)			
Log-additive	---	---	---	1.08 (0.97-1.21)	0.170	3446.6	3469.8

Supplementary Table 4. Different inheritance models analysis of the SNP rs2237895 between the NDM and T2DM group.

Model	Genotype	NDM	T2DM	OR (95% CI)	*P* value	AIC	BIC
Codominant	A/A	621 (48.1%)	509 (42.6%)	1.00	0.022	3442.9	3472
	C/A	552 (42.7%)	568 (47.6%)	1.26 (1.07-1.48)			
	C/C	119 (9.2%)	117 (9.8%)	1.20 (0.91-1.59)			
Dominant	A/A	621 (48.1%)	509 (42.6%)	1.00	0.006	3441	3464.2
	C/A-C/C	671 (51.9%)	685 (57.4%)	1.25 (1.06-1.46)			
Recessive	A/A-C/A	1173 (90.8%)	1077 (90.2%)	1.00	0.630	3448.2	3471.5
	C/C	119 (9.2%)	117 (9.8%)	1.07 (0.82-1.40)			
Overdominant	A/A-C/C	740 (57.3%)	626 (52.4%)	1.00	0.014	3442.5	3465.7
	C/A	552 (42.7%)	568 (47.6%)	1.22 (1.04-1.43)			
Log-additive	---	---	---	1.15 (1.02-1.30)	0.020	3443.1	3466.3

Supplementary Table 5. Different inheritance models analysis of the rs231362 between the NDM and T2DM group.

Model	Genotype	NDM	T2DM	OR (95% CI)	*P* value	AIC	BIC
	C/C	1032 (79.9%)	940 (78.7%)	1.00			
Codominant	C/T	241 (18.6%)	236 (19.8%)	1.07 (0.88-1.31)	0.780	3450	3479.1
	T/T	19 (1.5%)	18 (1.5%)	1.04 (0.54-2.00)			
Dominant	C/C	1032 (79.9%)	940 (78.7%)	1.00	0.490	3448	3471.3
	C/T-T/T	260 (20.1%)	254 (21.3%)	1.07 (0.88-1.30)			
Recessive	C/C-C/T	1273 (98.5%)	1176 (98.5%)	1.00	0.930	3448.5	3471.7
	T/T	19 (1.5%)	18 (1.5%)	1.03 (0.54-1.97)			
Overdominant	C/C-T/T	1051 (81.3%)	958 (80.2%)	1.00	0.490	3448	3471.3
	C/T	241 (18.6%)	236 (19.8%)	1.07 (0.88-1.31)			
Log-additive	---	---	---	1.06 (0.89-1.26)	0.510	3448	3471.3

Supplementary Table 6. Different inheritance models analysis of the rs5210 between the NDM and T2DM group.

Model	Genotype	NDM	T2DM	OR (95% CI)	*P* value	AIC	BIC
Codominant	A/A	368 (28.5%)	319 (26.7%)	1	0.420	3448.7	3477.8
	G/A	654 (50.6%)	602 (50.4%)	1.06 (0.88-1.28)			
	G/G	270 (20.9%)	273 (22.9%)	1.16 (0.93-1.46)			
Dominant	A/A	368 (28.5%)	319 (26.7%)	1	0.330	3447.5	3470.8
	G/A-G/G	924 (71.5%)	875 (73.3%)	1.09 (0.91-1.30)			
Recessive	A/A - G/A	1022 (79.1%)	921 (77.1%)	1	0.240	3447.1	3470.4
	G/G	270 (20.9%)	273 (22.9%)	1.12 (0.93-1.35)			
Overdominant	A/A - G/G	638 (49.4%)	592 (49.6%)	1	0.920	3448.5	3471.7
	G/A	654 (50.6%)	602 (50.4%)	0.99 (0.85-1.16)			
Log-additive	---	---	---	1.08 (0.96-1.21)	0.190	3446.8	3470

Supplementary Table 7. Different inheritance models analysis of the SNP rs5219 between the NDM and T2DM group.

Model	Genotype	NDM	T2DM	OR (95% CI)	*P* value	AIC	BIC
Codominant	C/C	462 (35.8%)	431 (36.1%)	1	0.720	3449.8	3478.9
	C/T	636 (49.2%)	572 (47.9%)	0.96 (0.81-1.14)			
	T/T	194 (15%)	191 (16%)	1.05 (0.83-1.34)			
Dominant	C/C	462 (35.8%)	431 (36.1%)	1	0.840	3448.4	3471.7
	C/T-T/T	830 (64.2%)	763 (63.9%)	0.98 (0.83-1.16)			
Recessive	C/C-C/T	1098 (85%)	1003 (84%)	1	0.500	3448	3471.3
	T/T	194 (15%)	191 (16%)	1.08 (0.87-1.34)			
Overdominant	C/C-T/T	656 (50.8%)	622 (52.1%)	1	0.490	3448	3471.3
	C/T	636 (49.2%)	572 (47.9%)	0.95 (0.81-1.11)			
Log-additive	---	---	---	1.01 (0.90-1.14)	0.830	3448.4	3471.7

Supplementary Table 8. The genotype of rs2237897 with glucose and lipid metabolic parameters in T2DM group

	C/C	C/T	T/T	*P* value
TC(mmol/L)	4.842±1.079	4.794±1.085	4.781±1.012	0.733
TG(mmol/L)	2.484±2.234	2.431±2.013	2.468±2.031	0.924
HDL(mmol/L)	1.098±0.291	1.084±0.264	1.111±0.304	0.563
LDL(mmol/L)	2.818±0.995	2.778±0.926	2.745±1.037	0.697
GLU(mmol/L)	7.993±1.054	7.777±2.325	7.961±2.563	0.361
HbA1C(%)	9.282±2.921	8.688±2.674	8.949±2.817	0.003
